# Single nucleotide polymorphism-based analysis of the genetic structure of the Min pig conserved population

**DOI:** 10.5713/ab.21.0571

**Published:** 2022-06-30

**Authors:** Fanbing Meng, Jiancheng Cai, Chunan Wang, Dechang Fu, Shengwei Di, Xibiao Wang, Yang Chang, Chunzhu Xu

**Affiliations:** 1 College of Life Science, Northeast Agricultural University, Harbin 150030, China; 2 Lanxi Breeding Farm, Lanxi 151500, China; 3 College of Animal Science and Technology, Northeast Agricultural University, Harbin 150030, China

**Keywords:** Genetic Diversity, Molecular Pedigree, Population Structure, Single Nucleotide Polymorphism (SNP) Chip

## Abstract

**Objective:**

The study aims to uncover the genetic diversity and unique genetic structure of the Min pig conserved population, divide the nucleus conservation population, and construct the molecular pedigree.

**Methods:**

We used KPS Porcine Breeding Chip v1 50K for SNP detection of 94 samples (31♂, 63♀) in the Min pig conserved population from Lanxi breeding Farm.

**Results:**

The polymorphic marker ratio (PN), the observed heterozygosity (Ho), and the expected heterozygosity (He) were 0.663, 0.335, and 0.330, respectively. The pedigree-based inbreeding coefficients (F_PED_) was significantly different from those estimated from runs of homozygosity (F_ROH_) and single nucleotide polymorphism (F_SNP_) based on genome. The Pearson correlation coefficient between F_ROH_ and F_SNP_ was significant (p<0.05). The effective population content (Ne) showed a continuously decreasing trend. The rate of decline was the slowest from 200 to 50 generations ago (r = 0.95), then accelerated slightly from 50 to 5 generations ago (1.40<r<1.50) and increased significantly in the last 5 generations (r = 2.6). According to the composition of Chinese lineage, we separated the nucleus conservation population (81 individuals) and the candidate conservation population (13 individuals) of Min pig, then the nucleus conservation population of Min pig was divided into 9 families by genomic information matrix.

**Conclusion:**

Our study indicated that the genetic diversity of the Min pig conserved population was inadequate. Due to the introgression of European commercial pig breeds and the unscientific breeding process, it is necessary to construct the molecular pedigree of the nucleus conservation population for the Min pig.

## INTRODUCTION

Animal domestication is a groundbreaking event that has profoundly influenced human history [1]. The pig (*Sus scrofa*) was domesticated independently in Europe and Asia approximately 10,000 years ago [2,3]. As the domestication center of pigs in East Asia, China with two origin centers in the middle and northeast of the Yellow River [4], possesses abundant domestic pig germplasm resources, and more than one-third of global pig breeds are grown in China [5]. The indigenous pig breeds in China, range over diverse geographical conditions, various climates, and human environments, have undergone thousands of years of natural and artificial selection, forming many unique and excellent traits [6].

With the construction of the Middle East railway at the beginning of the 20th century, China has witnessed an unprecedented development of the pig industry over the past hundred years. There is no denying that an important part of this development is based on the continued importation of European commercial pigs. These commercial breeds occupy a dominant position in pig industry of China [7]. Because of their excellent performance in growth rate, lean rate, and feed conversion efficiency, they undoubtedly poses a great threat to Chinese indigenous pigs, leading to a sharp decline in the popularity of most Chinese indigenous breeds, resulting in 20 breeds becoming extinct [5]. To solve this alarming problem, Chinese government has launched a National Conservation Program for 42 indigenous pig breeds [1].

Min pig has excellent characteristics, including high reproductive rate, cold resistance, and rough feeding resistance, and has been selected as one of the eight high-quality pig breeds of the world. The importation of European commercial pigs has also led to a sharp decrease in the population of Min pigs [5]. As a result, Min pig has been included in the national protection plan, and a national conservation farm has been established to protect the fine traits of Min pig to a certain extent. However, traditional conservation is highly dependent on pedigree [8], and inaccurate pedigree information will affect the reliability of studies using pedigree information such as gene mapping and genome selection. Therefore, it is necessary to evaluate the current status of genetic diversity in the Min pig conserved population and reveal its unique fine population structure formation mechanism in the global population background.

To establish a reliable conservation strategy for farmers and maintain genetic diversity to the utmost by avoiding severe inbreeding within each family, an in-depth investigation of the genetic structure of Min pig conserved population is required, and identifying the possible admixture events between different breeds. In this study, based on the data of KPS Porcine Breeding Chip v1 50K, we selected 94 individuals of the Min Pig conserved population as the research object and 130 individuals of 13 Eurasian pig breeds as the research background to analyze the genetic diversity and genetic structure of the Min pig conserved population, determine its lineage compositions, divide the nucleus conservation population, and construct the molecular pedigree. These findings will provide novel insight into scientific conservation and rational breeding strategy for the Min pig.

## MATERIALS AND METHODS

### Animal care

All the procedures and animal works were conducted according to the guidelines for the care and use of experimental animals established by the Ministry of Agriculture and villages of China and were approved by the Ethics and Experimental Animal Committee of Northeast Agricultural University, China.

### Sample collection

All the individuals (31♂, 63♀) from the same generation of the Min pig conserved population were selected from the National Conservation Farm for Min pig in Lanxi County, Heilongjiang Province, China. We traced 4 generations of pedigree records of these individuals.

### DNA extraction and identification

Ear tissue samples were collected and placed in cryopreserve tubes for rapid preservation in liquid nitrogen. Genomic DNA was extracted by phenol-chloroform extraction method and stored at −20°C. The quality of DNA was determined by ultraviolet spectrophotometry (NanoDrop 2000; Thermo Scientific, ShangHai, China), and gel electrophoresis (DYY-6C & DYCP-31BN; Beijin Liuyi, Beijing, China). The DNA with OD value between 1.8 and 2.0 was diluted to a final concentration of 20 ng/μL.

### Genotyping and data quality control

The qualified DNA samples were genotyped using KPS Porcine Breeding Chip v1 50K (Compass Biotechnology Co., Ltd., Beijing, China), which is a chip designed based on the application of genome selection breeding. The chip meets the requirements of genome selection breeding for the chip that “involving as many traits as possible, distribute evenly on the genome, which has high polymorphism and strong specificity”. In the chip design, the single nucleotide polymorphism (SNP) variation loci related to the growth, reproduction and meat quality traits of Chinese local pig breeds were obtained by resequencing technology. The credibility of SNP loci was scored according to the requirements of minor allele frequency (MAF) and genome average distribution. Finally, the chip was customized by Illumina optical fiber microbead technology, and a total of 51,315 SNP was contained.

To reveal the fine population structure of Min pig from a global perspective, we extracted the common sites of KPS porcine breeding chip v1 (including Min pig, Hebao pig, Dapulian pig, and Laiwu pig) and Illumina 60K SNPs chip (including 3 European commercial pigs, 1 Asian wild boar and 6 Asia local pigs) according to the chromosome information and physical location information of SNPs loci, which were common SNP sites in pigs. A total of 23,708 sites were extracted for follow-up analysis [9,10]. The above quality control on the combined SNP data was performed by PLINK v1.90 software [11]. Quality control of the genotypic data was conducted with the following criteria: i) retaining the SNPs located on autosomes; ii) filtering out individuals with a call rate below 0.90; iii) removing the SNPs with a call rate less than 0.90; iv) removing the SNPs with a MAF below 0.01; and v) removing the SNPs with a Hardy Weinberg equilibrium p value less than 1×10^−6^.

### Genetic diversity analyses

Based on the genome-wide SNP quality control data, we used PLINK v1.90 software to calculate the MAF, the percentage of polymorphic marker ratio (PN), the observed heterozygosity (Ho) and the expected heterozygosity (He). We estimated the effective population content (Ne) based on the level of linkage disequilibrium [12,13]:


$$\text{Ne}=(1/4\text{c})\times (1/{\text{r}}^{2}-1)$$

where r^2^ is estimated by the linkage disequilibrium decline model, Ne represents the effective population content, and c represents the Morgan distance between SNP sites [14]. In this study, inter-SNP distances (kb) were binned into the following classes: 0.5, 1, 2, 5, 10, and 100 Mb, the Ne of the Min pig conserved population in the corresponding 200, 50, 20, 10, 5 generations and the current generation was estimated, where 1 cm is approximately equal to 1 Mb.

### Runs of homozygosity detection

Runs of homozygosity (ROH) for each individual were estimated using “- homozyg” of PLINK v1.90 software. The default parameter –homozyg were used to define ROH and the following criteria were chosen: i) 50 SNPs were contained in each sliding window; ii) an ROH consisted of no less than 30 consecutive SNPs; iii) the density should be higher than one SNP per 1,000 kb; iv) SNPs with missing genotypes less than one and with a heterozygous genotype less than one were allowed in an ROH due to genotyping error. All homozygous segments filtered were classified into three length classes: 1 to 5, 5 to 10, and >10 Mb, identified as ROH 1 to 5 Mb, ROH 5 to 10 Mb, and ROH>10 Mb, respectively.

### Pedigree and genomic inbreeding coefficients

Pedigree-based inbreeding coefficients (F_PED_) for all pigs were estimated using R package pedigree. Then, genomic inbreeding coefficient (F_ROH_) of all individuals was estimated by PLINK v1.90 software, which was calculated according to the following formula [15]:


$${\text{F}}_{\text{ROH}}={\text{L}}_{\text{ROH}}/{\text{L}}_{\text{AUTO}}$$

Where L_ROH_ was defined as the total length of the genome covered by all ROH segments for each individual, and L_AUTO_ was the length of the sequenced genome, which equaled 2,450,462.292 kb in this research. For each animal, four ROH estimates were calculated based on lengths from sequence data as the proportion of its genome: ROH>10 Mb (F_ROH>10 Mb_), 5 to 10 Mb (F_ROH 5–10 Mb_), 1 to 5 Mb (F_ROH 1–5 Mb_), and ROH>1 Mb (F_ROH>1 Mb_), corresponding to 5 generations, 5 to 10 generations, 10 to 50 generations, and 50 generations, respectively. In addition, SNP-based inbreeding coefficients were estimated using the option –ibc from the GCTA software [16]. The inbreeding coefficients obtained by the methods were compared using Pearson’s correlation.

### Population structure analysis

#### Admixture analysis

A total of 224 pigs from Asian wild boar (WB), 9 Chinese indigenous pig breeds, which contain Erhualian (EHL), Jinhua (JH), Bama Xiang (BMX), Hebao (HB), Dapulian (DPL), Laiwu (LAW), Ganxi (GX), Rongchang (RC), Tibetan Pig (ZZG), and 3 European commercial pig breeds which contain Duroc (DRC), Landrace (LR) and large White (LW) were used as the genetic background for population analysis. The genetic structure of Min pig was analyzed by ADMIXTURE v1.30 [17]. To reduce the effect of ascertainment bias, we used the genotype data of the pruned 28,176 SNPs with LD (r^2^) values of less than 0.5. We randomly selected 10 pigs from each population for mixed analysis, and the best K value was determined by cross-validation error. Finally, the internal R script was used to visually infer the population structure.

### Selection of the nucleus conservation

The ancestral pedigree composition of all Min pigs was subdivided by ADMIXTURE analysis. When K = 2, clarified the proportion, average and standard deviation of individuals Chinese lineage in the Min pig conserved population. To select the nucleus conservation and the candidate conservation population, we divided the proportion values into four categories, including those greater than the mean value (Min1), between the mean and the mean minus one standard deviation (SD) (Min2), between the mean minus two SDs (Min3), and less than the mean minus two SDs (Min4).

### Molecular pedigree construction of the nucleus conservation

#### Kinship analysis

The coefficient of kinship is not only the key parameter to evaluate the genetic structure of population, but also the basis for selection and variety protection in breeding. In this study, the R package pedigree was used to construct the kinship matrix (A matrix) based on the traditional pedigree. The PLINK v1.90 GCTA [16] was used to construct the SNP-based kinship matrix (G matrix). We jointly constructed the kinship matrix (H matrix) using the one-step method (single-step) through the pedigree and genome information. Each matrix calculated the kinship between the nucleus conservation of Min pig. Finally, we calculated the Pearson correlation coefficient based on the traditional pedigree and the genome information matrix.

#### Phylogenetic analysis

Male individuals are the main carrier for the pedigree construction of the conserved population. Firstly, the male individuals were analyzed separately for phylogenetic analysis, and the kinship among them was identified. In accordance with the principle of selection and breeding the three generations are not related in the process of genetic breeding of the population, the family is divided according to the standard that the kinship coefficient between individuals is less than 0.0625. Based on the longest distance method, the R package hclust was used to analyze the male individuals of A, G, and H matrices respectively, then determined the most suitable number of families. The genetic distance (D) among all individuals of the conserved population was calculated by the following formula:


(D)=1-r

where r is the coefficient of kinship among individuals. Finally, the molecular pedigree structure diagram of the nucleus conservation of Min pig was visualized using R package ggtree.

## RESULTS

### SNP typing and quality control

We genotyped and quality controlled 94 Min pigs with the KPS Porcine Breeding Chip v1 50K. A total of 51,315 SNPs loci were detected, and the sample call rate was 98.38% to 98.84%, with an average of 98.66%. Among that, 44,739 SNPs were identified as autosomal. A total of 1,383 SNPs were deleted because the call rate was less than 0.90, 11,202 SNPs were deleted because the MAF value was less than 0.01, and 460 SNPs were filtered because they significantly deviated from the H-W equilibrium with the p value less than 1×10^–6^. Finally, a total of 31,694 SNPs data that met high-quality control standards were obtained. After integrating SNP data with Illumina 60K (52,556 SNPs), 23,708 SNPs data from 224 pigs obtained by the same quality control standard were used for the following analyses.

### Genetic diversity analyses

**Figure 1:**
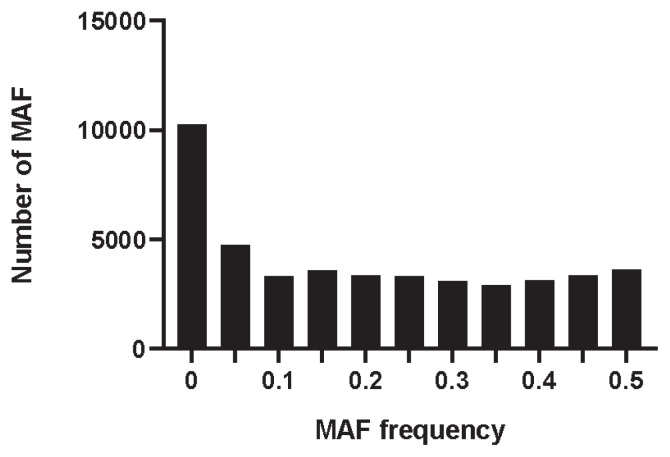
Distribution of minor allele frequency in Min pig based on SNP. The x-axis denotes the MAF frequency statistics, whereas y-axis represents the number of MAF. SNP, single nucleotide polymorphism; MAF, minor allele frequency.

The MAF distribution of all SNPs was calculated by PLINK v1.90 software. As shown in Figure 1, the MAF was distributed in each interval, with an average of 0.185. The percentage of PN in the Min pig conserved population was 0.663, the observed heterozygosity (Ho) was 0.335, and the expected heterozygosity (He) was 0.330. The Ho was slightly higher than the He.

**Figure 2:**
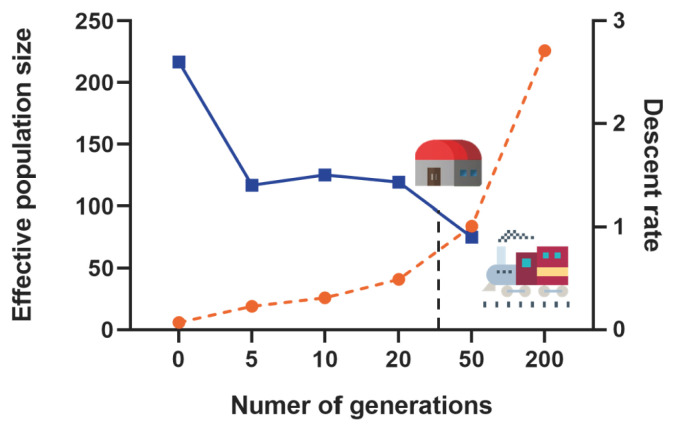
Effective population size estimation along 200 generations. The orange dotted line represents the number of generations, the blue dotted line represents the descent rate of generations, and the black dotted line indicates the establishment time of the Min pig conservation farm.

Based on the autosomal genome information of the Min pig conserved population, we calculated the linkage disequilibrium (r^2^) between all SNPs pairs of each chromosome, and then evaluated the effective population content (Ne). In this study, a total of 2,514 million pairs of SNPs linkage disequilibrium estimates were obtained, inter-SNP distances (kb) were binned into the following classes: 0.5, 1, 2, 5, 10, and 100 Mb, and historical Ne was estimated from 200 to 0 generations ago. The Ne of the Min pig conserved population showed a continuously decreasing trend. The rate of decline was the slowest from 200 to 50 generations ago (r = 0.95), then accelerated slowly from 50 to 5 generations ago (1.40<r<1.50) and increased significantly in the last 5 generations (r = 2.6) (Figure 2).

### Genomic distribution of runs of homozygosity

**Figure 3:**
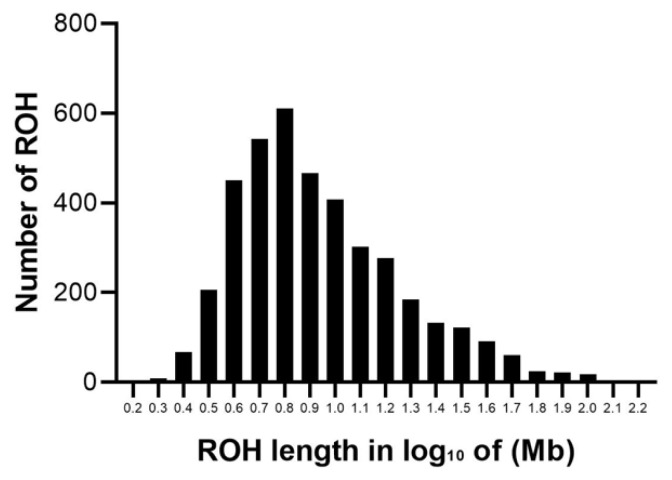
Distribution of ROH in different lengths (Mb). The values of length in Mb were transformed in log10. The y-axis represents the content of ROH with different lengths. ROH, runs of homozygosity.

**Figure 4:**
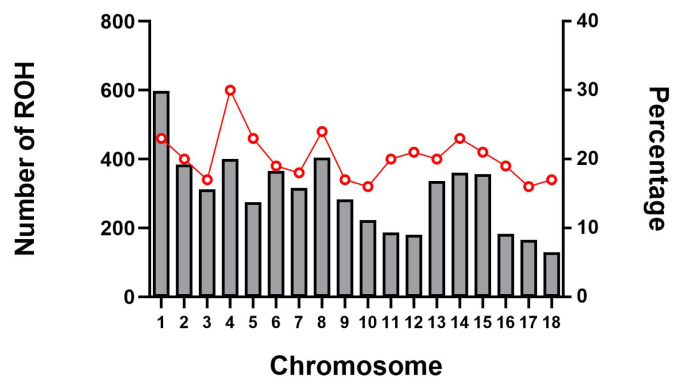
Number of ROH longer than 1 Mb per chromosome (bars) and average percentage of each chromosome covered by ROH (red line). The x-axis represents the chromosomal location, whereas left y-axis represents the number of ROH on each chromosome and the right y-axis represents the percentage content of ROH coverage on each chromosome. ROH, runs of homozygosity.

The abundance and genomic distribution of ROH provides efficient information about the demographic history of livestock species. A total of 5,456 ROH were obtained from 94 individuals by PLINKv1.90 software, with an average of 58 ROH per individual. The total length of ROH was 507.83 Mb, the mean ROH length was 9.31 Mb, and all ROH segments account for 22.05% of the whole genome. The distribution of ROH according to length is shown in Figure 3. For chromosomes, the number of ROH per chromosome and the percentage of chromosomes covered by ROH are shown in Figure 4. The highest number of ROH per chromosome was on SSC1 (598 segments), accounting for 23% of the chromosome, whereas the lowest was on SSC18 (129 segments), accounting for 17% of the chromosome. Among them, the longest ROH segment was on SSC13 (132.9 Mb), containing 2,474 SNPs, and the shortest ROH segment was on chromosome SSC15 (1.13 Mb), containing 55 SNPs.

The descriptive statistics of ROH number and length by classes are given in Table 1. Among the different classes of ROH segments, the majority were short ROH segments (1 to 5 Mb), accounting for 45.16% of the total number of ROH segments, ROH segments (5 to 10 Mb) made up 29.80% of the total ROH length, the number of long ROH segments was the lowest, but it still had a coverage ratio of 25.04%.

### Pedigree and genomic inbreeding coefficients

The inbreeding coefficient of the Min pig conserved population estimated by pedigree, ROH of different lengths, and the SNP are shown in Table 2. The average inbreeding coefficient estimated by pedigree information was F_PED_ = 0.015 ±0.032, and the average inbreeding coefficients estimated by ROH of different lengths were F_ROH > 10 Mb_ = 0.133±0.038, F_ROH 5–10 Mb_ = 0.049±0.011, F_ROH 1–5 Mb_ = 0.037±0.007, F_ROH>1 Mb_ = 0.220±0.037. And the average inbreeding coefficient estimated by SNP was F_SNP_ = −0.014±0.040. Though there were obvious differences among F_PED_, F_ROH_, and F_SNP_, there was also a certain correlation between them. Among them, the Pearson correlation between F_ROH>1 Mb_ and F_SNP_ was the highest and significant (p<0.05), while the Pearson correlation between F_ROH>1 Mb_, F_SNP_, and F_RED_ was not significant (p>0.05).

### Population structure analysis

#### Admixture analysis

**Figure 5:**
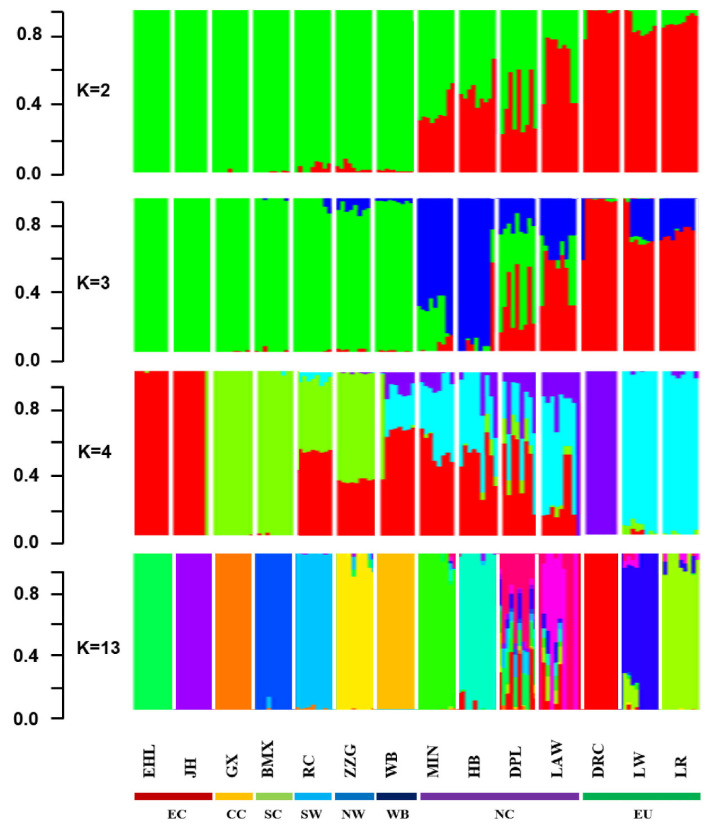
The ancestry compositions of 14 Eurasian breeds and Chinese wild boar that were uncovered by ADMIXTURE with the assumed number of ancestries from 2 to 4 and 13. Each color represents one ancestral cluster. Abbreviations: EHL, Erhualian; JH, Jinhua; GX, Ganxi; BMX, Bamaxiang; RC, Rongchang; ZZG, Gansu Tibet; Min, Min; HB, Hebao; DPL, Dapulian; Laiwu, Laiwu; DRC, Duroc; LW, Large White; LR, Landrace.

For investigating the historical admixture pattern of Min pig, the ancestral lineage compositions analysis was carried out on 140 samples from 14 Eurasian pig breeds (including 10 Min pigs) in the global background by ADMIXTURE v1.30. The results showed that Min pig had obvious signatures of introgression with European commercial pig breeds (K = 2, 3, 4). When K = 13 that represented the optimal number of assumed ancestors by cross-validation error test, a certain proportion of European ancestries were still evidenced in Min pig (Figure 5).

### Selection of the nucleus conservation

**Figure 6:**
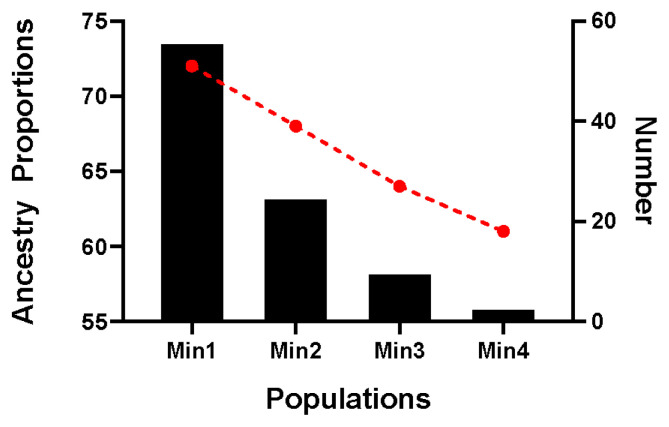
Distribution of Chinese lineage compositions in 94 Min pigs. The red line chart represents the proportion of Chinese lineage compositions, and the black column chart represents the number in each population.

The proportion of Chinese lineage in Min Pig conserved population was analyzed by ADMIXTURE v1.30. The results showed that the average proportion of Chinese lineage in Min pig population was 0.70±0.04 (Supplementary Table S1). According to the proportion of Chinese lineage, the nucleus conservation (greater than mean minus one SD) and candidate conservation population (less than mean minus one SD) were 81 and 13, respectively (Figure 6).

### Molecular pedigree construction of the nucleus conservation

#### Kinship analysis

Based on pedigree, genomic markers and their combination, A matrix, G matrix and H matrix were constructed respectively, and the kinship coefficients between 3,240 pairs in 81 samples of the Min pig conserved population were calculated. The results showed that the average kinship coefficient of A matrix based on pedigree was 0.02, and those of G matrix and H matrix based on genomic markers were both −0.03. We also calculated the Pearson correlation coefficients among A matrix and G matrix and H matrix, which were 0.51, 0.53 and 1 respectively, and the correlations were all significant (p<0.05) (Table 3).

#### Phylogenetic analysis

**Figure 7:**
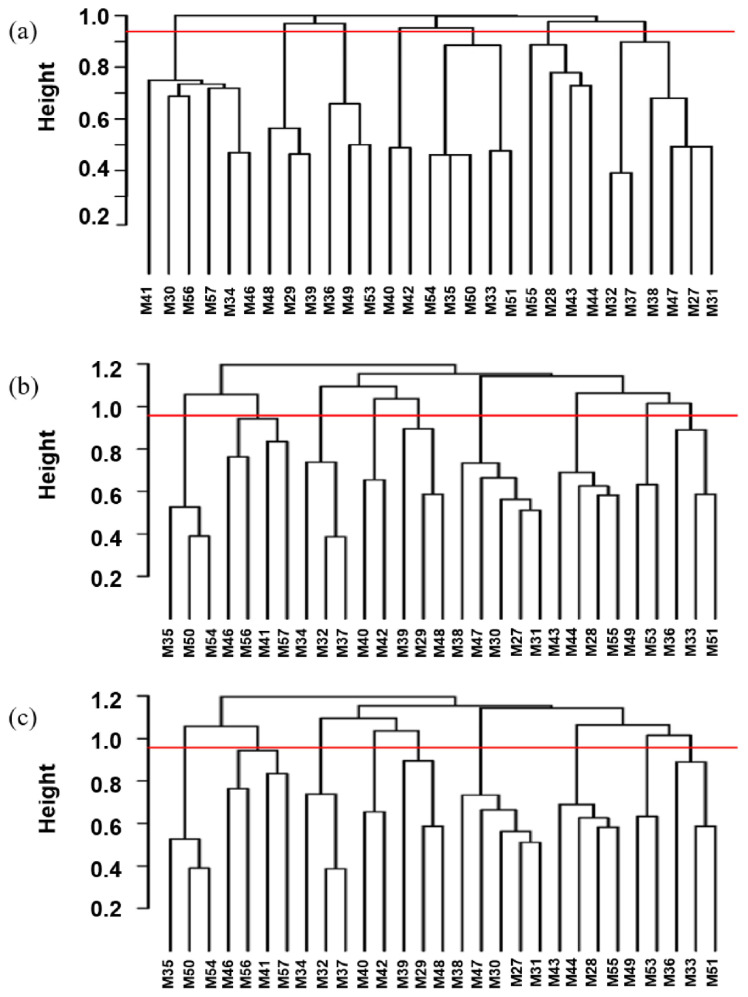
Distribution of the boar pedigrees based on (a) A matrix, (b) G matrix, (c) H matrix. The x-axis represents the boar individuals, and the y-axis represents the kinship coefficient. Above the red line represents a kinship coefficient greater than 0.0625.

**Figure 8:**
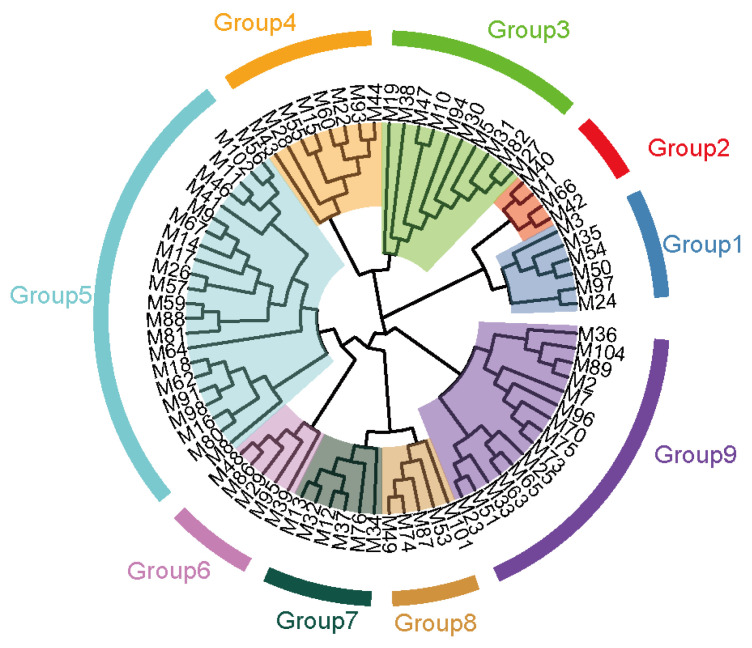
Distribution Map of the nucleus conservation population of Min pig. Each color represents one family, and the blue star represents the boar of each family.

Based on the longest distance method, the R package hclust was used to analyze A, G, and H matrices of the male individuals respectively. The results are shown in Figure 7A, B, and C. Individuals with kinship coefficient less than 0.0625 were divided into the same family. Based on the A matrix, the nucleus conservation of Min pig was divided 7 families, and based on the G matrix and H matrix, it was divided into 9 families, the number of male individuals in each family was relatively evenly distributed. According to the division standard of 9 families, the genetic distance among all individuals of the Min pig conserved population was constructed by R package ggtree (Figure 8).

## DISCUSSION

### Genetic diversity of the Min pig conserved population

The genetic diversity analysis is very important for the protection of the population. Through different indicators of genetic diversity, we can evaluate the current state of the population. In this study, the whole-genome high-density SNP markers were used to evaluate the conservation status of Min pig for the first time. The heterozygosity analysis results elucidated that the expected heterozygosity (He) of the Min pig conserved population was 0.330, which was lower than Jinhua pig (He = 0.429) [18], Laiwu pig (He = 0.380) [19], Erhualian pig (He = 0.378) and Meishan pig (He = 0.382) [20]. The low expected heterozygosity of the Min pig conserved population indicates that many SNPs loci tended to be pure and may be undergoing inbreeding. In addition, the observed heterozygosity (Ho) of the Min pig conserved population was 0.335, which was slightly higher than that of He, indicating that there may be differentiation or introduction of foreign lineage in the history of the evaluated population.

Effective population size (Ne), as another important indicator of genetic diversity, plays an important role in the process of animal genetic breeding [21]. The result of this study showed that the decline rate of the Ne of Min pig was the slowest from 200 to 50 generations ago, then accelerated slowly, and increased significantly in the last 5 generations. We indicated that many European commercial pig breeds have been introduced since the construction of the Middle East Railway in the early 20th century, resulting in genetic introgression of Min pig. With the establishment of the Min pig National Conservation Farm in the 1970s, genetic introgression was blocked. However, due to the founder effect, the Ne of small-scale Min pig population has declined slowly since the 50th generation. In the past 5 generations, the inbreeding of Min pig may have increased due to poor management of breeding farms or other factors, which has led to a sharp increase in the decline rate of their Ne. In addition, some studies have confirmed that if the Ne value of a small population is less than 65, it means that the population is endangered [22]. The Ne of the Min pig conserved population is gradually decreasing, with a Ne value of 19 in the last 5 generations, which is in a threatened state. In the long term, it may lead to increase inbreeding and even genetic drift in the population. Therefore, the conservation farm should consider the ratio of male and female individuals and the lineage compositions of pigs to ensure the dynamic balance of the conservation population as much as possible.

Defining the inbreeding coefficient of the population also plays an important role in conservation work. The reduction of genetic diversity can be avoided by controlling inbreeding. Traditional estimation of inbreeding coefficient is based on pedigree, however, since it is incomplete, it is more accurate to use genomic information to estimate the inbreeding coefficient [23]. Among them, ROH has become a popular method to evaluate the inbreeding of populations [24,25]. Different length fragments of ROH can distinguish the inbreeding history of ancient and recent, respectively [26,27]. In this study, the FROH value of the Min pig conserved population has continued to increase from 50 generations ago, which directly reflects that the Min pig conserved population is undergoing inbreeding and has had an impact on the genome of Min pig, which should be taken seriously. Among them, the F_ROH 1–5 Mb_ value was the lowest, which could also reflect the possible genetic introgression of exotic pig breeds in the process of domestication. The overall inbreeding coefficient (F_ROH>1 Mb_) value was high, which may be due to the genetic relationship among individuals of Min pig, and the influence of pedigree effect on inbreeding analysis.

### Molecular pedigree construction of the nucleus conservation

Since the beginning of the 20th century, a large number of European commercial pigs have been introduced into China, crossed with indigenous breeds and pose a serious threat to Chinese indigenous pig breeds [28]. In this study, the results of Admixture analysis clearly showed that the European commercial pigs had historically flowed to the Min pig conserved population, which led to the mixed European lineage in Min pig. Among the 94 Min pigs, 81 Min pigs have a Chinese lineage ratio greater than mean minus one SD (66.0%). It is suggested that 81 Min pigs should be taken as the nucleus conservation population, then the remaining 13 Min pigs whose Chinese lineage ratio less than mean minus one SD (66.0%) are suggested as the candidate conservation population. During the conservation process, family rotation mating is adopted to ensure 90% genetic diversity of livestock. Future research will focus on the effects of the introgression of European commercial pig breeds on the traits of Min pig, define the domestication history of Min pig, further explore the list of candidate genes for introgression, and deepen the understanding of the genetic background of the unique trait mechanism of Min pig.

In the breeding process, there will inevitably be errors and deficiencies in the record of pedigree. Especially for the conservation farms, it may be difficult to evaluate the true kinship between individuals in the subsequent analysis, resulting in a decrease in the accuracy of analysis [29]. Meanwhile, since to the pedigree in this study can only be traced back to the effective information of 4 generations of the Min pig conserved population, the true kinship between individuals cannot completely and truly represented. The genomic kinship matrix constructed by whole genome SNP markers can more truly reflect the individual kinship [30] when the marker density is appropriate. Therefore, while constructing the A matrix based on pedigree, we also adopted the strategy of constructing the molecular pedigree G matrix and the pedigree -genome joint matrix H matrix based on the genomic information and calculated the correlation coefficient between different matrices. The results showed that the A matrix based on pedigree and the G matrix and H matrix based on the genome are strongly correlated and extremely significant, indicating that the constructed molecular pedigree is more accurate than incomplete pedigree information, and the molecular pedigree can be divided into 9 families, in which the distribution of male individuals is relatively uniform, and will lay a foundation for further breeding of rational utilization of families.

## CONCLUSION

The genetic structure of the Min pig conserved population was analyzed by genome-wide SNP chip data. We found that the level of genetic diversity of the Min pig conserved population was inadequate. In the past 5 generations, the inbreeding degree of the conserved population increased significantly, and the content of the effective population decreased dramatically. Due to the introgression of European commercial pig breeds, and the unscientific breeding process, it is necessary to construct the molecular pedigree of the nucleus conservation population for Min pig. In summary, our study findings not only contribute to an in-depth understanding of the population genetic characteristics of Min pig, but also provide powerful instruction for conservation to reduce the risk of decline.

## Figures and Tables

**Table 1:** Descriptive statistics of runs of homozygosity (ROH) number and length (in Mb) by ROH length class (ROH 1 to 5 Mb, ROH 5 to 10 Mb, ROH>10 Mb and total) in Min pig

ROH length (Mb)	ROH number	Percent (%)	Mean length (Mb)	Standard deviation	Genome coverage (%)
1 to 5	2,464	45.16	3.48	0.81	3.72
5 to 10	1,626	29.80	6.92	1.40	4.89
>10	1,366	25.04	22.67	15.10	13.44
Total (>1)	5,456	100	9.31	10.94	22.05

**Table 2:** Descriptive statistics and correlation coefficients between the inbreeding coefficient based on pedigree (F_PED_), ROH (F_ROH>10 Mb_, F_ROH 5–10 Mb_, F_ROH 1–5 Mb_, F_ROH>1 Mb_) and SNP-by-SNP (F_SNP_)

Inbreeding coefficient	Mean	SD	F_ROH>10 Mb_	F_ROH 5–10 Mb_	F_ROH 1–5 Mb_	F_ROH>1 Mb_	F_SNP_	F_PED_
F_ROH>10 Mb_	0.133	0.038	1					
F_ROH 5–10 Mb_	0.049	0.011	−0.110	1				
F_ROH 1–5 Mb_	0.037	0.007	−0.230^*^	0.050^*^	1			
F_ROH>1 Mb_	0.220	0.037	0.850^*^	0.200^*^	−0.010	1		
F_SNP_	−0.014	0.040	0.220^*^	0.280^*^	0.080	0.410^*^	1	
F_PED_	0.015	0.032	−.220^*^	−0.140	−0.020	−0.020	−0.080	1

SNP, single nucleotide polymorphism; SD, standard deviation.

* Significantly different p<0.05.

**Table 3:** Kinship coefficient analysis among A matrix, G matrix and H matrix

Name	Mean	SD	A matrix	G matrix	H matrix
A matrix	0.02	0.11	1		
G matrix	−0.03	0.12	0.51^*^	1	
H matrix	−0.03	0.12	0.53^*^	1^*^	1

SD, standard deviation.

* Significantly different p<0.05.
