# Supplementary material for: Single nucleotide polymorphism-based analysis of the genetic structure of the Min pig conserved population
Source: Anim Biosci. 2022 Jun 30;35(12):1839–49. doi: 10.5713/ab.21.0571 (PMC9659443; doi:10.5713/ab.21.0571)
Supplement: Supplementary file 1 [file ab-21-0571-suppl.pdf]

Min1		Min2		Min3		Min4	
M1	70%	M7	66%	M6	63%	M69	62%
M2	74%	M12	66%	M20	63%	M85	61%
M3	71%	M16	67%	M45	65%	M99	59%
M5	72%	M19	67%	M52	64%		
M9	72%	M35	67%	M58	63%		
M13	71%	M36	69%	M68	65%		
M14	78%	M37	67%	M77	63%		
M15	71%	M41	69%	M79	64%		
M17	74%	M46	67%	M92	65%		
M18	71%	M49	69%	M90	65%		
M22	75%	M50	67%				
M23	74%	M53	69%				
M26	76%	M56	67%				
M28	71%	M64	66%				
M29	72%	M63	66%				
M30	72%	M73	68%				
M32	70%	M76	69%				
M33	74%	M80	66%				
M34	71%	M82	66%				
M38	71%	M31	69%				
M39	74%	M94	69%				
M40	76%	M96	68%				
M42	71%	M98	69%				
M43	74%	M101	69%				
M44	77%	M102	68%				
M47	70%						
M48	72%						
M51	75%						
M54	71%						
M55	74%						
M57	72%						
M59	72%						
M60	72%						
M62	70%						
M65	71%						
M66	73%						
M67	71%						
M70	70%						
M74	75%						
M75	72%						
M86	76%						
M87	71%						
M91	71%						
M81	71%						
M89	75%						
M93	78%						
M95	72%						
M97	75%						
M104	75%						
M8	70%						
M10	70%						
M11	70%						

M24	70%
M25	70%
M27	70%
M88	70%
